# Training general practitioners to improve evidence-based drug treatment of patients with heart failure: a cluster randomised controlled trial

**DOI:** 10.1007/s12471-020-01487-x

**Published:** 2020-09-30

**Authors:** M. J. M. Valk, A. W. Hoes, A. Mosterd, M. A. Landman, N. P. A. Zuithoff, B. D. L. Broekhuizen, F. H. Rutten

**Affiliations:** 1 Julius Centre for Health Sciences and Primary Care, University Medical Centre Utrecht, Utrecht, The Netherlands; 2 Department of Cardiology, Meander Medical Centre, Amersfoort, The Netherlands; 3 Leusden, The Netherlands

**Keywords:** Heart failure, Drug treatment, Primary care, Health status, Survival, Hospitalisation

## Abstract

**Aims:**

To assess whether a single training session for general practitioners (GPs) improves the evidence-based drug treatment of heart failure (HF) patients, especially of those with HF with reduced ejection fraction (HFrEF).

**Methods and results:**

A cluster randomised controlled trial was performed for which patients with established HF were eligible. Primary care practices (PCPs) were randomised to care-as-usual or to the intervention group in which GPs received a half-day training session on HF management. Changes in HF medication, health status, hospitalisation and survival were compared between the two groups. Fifteen PCPs with 200 HF patients were randomised to the intervention group and 15 PCPs with 198 HF patients to the control group. Mean age was 76.9 (SD 10.8) years; 52.5% were female. On average, the patients had been diagnosed with HF 3.0 (SD 3.0) years previously. In total, 204 had HFrEF and 194 HF with preserved ejection fraction (HFpEF). In participants with HFrEF, the use of angiotensin-converting enzyme inhibitors/angiotensin receptor blockers decreased in 6 months in both groups [5.2%; (95% confidence interval (CI) 2.0–10.0)] and 5.6% (95% CI 2.8–13.4)], respectively [baseline-corrected odds ratio (OR) 1.07 (95% CI 0.55–2.08)], while beta-blocker use increased in both groups by 5.2% (95% CI 2.0–10.0) and 1.1% (95% CI 0.2–6.3), respectively [baseline-corrected OR 0.82 (95% CI 0.42–1.61)]. For health status, hospitalisations or survival after 12–28 months there were no significant differences between the two groups, also not when separately analysed for HFrEF and HFpEF.

**Conclusion:**

A half-day training session for GPs does not improve drug treatment of HF in patients with established HF.

**Electronic supplementary material:**

The online version of this article (10.1007/s12471-020-01487-x) contains supplementary material, which is available to authorized users.

## What’s new?

- Heart failure (HF) management has improved substantially over the last two decades. Combination of prescribed diuretics, angiotensin-converting enzyme inhibitors and beta-blockers has substantially improved the prognosis mainly of patients with HF with reduced ejection fraction. However, earlier studies have shown that HF management in primary care is far from optimal with underprescription of the aforementioned drugs.
- A half-day training session for general practitioners does not improve drug prescription in patients with established HF.
- Other interventions, such as a multidisciplinary approach, should be considered in primary care for optimising HF drug treatment in patients with stable HF.

## Introduction

Heart failure (HF) is an increasing healthcare problem worldwide, and a multidisciplinary approach with a general practitioner (GP) in the healthcare team is considered optimal [1]. HF management has improved substantially over the last two decades, mainly for patients with HF with a reduced ejection fraction (HFrEF) with inhibition of the renin-angiotensin system and sympathetic nervous system [2, 3].

For patients with HF and a preserved ejection fraction (HFpEF), to date no drugs have been shown to clearly improve prognosis. Diuretics are helpful for fluid status management and thus reduce symptoms of fluid overload in HFpEF. Next, optimal blood pressure management is recommended and, in the case of tachycardia, optimal rate control or rhythm correction. Moreover, optimal management of comorbidities is important [3]. The search for novel treatment options for HFpEF patients is still ongoing [4–6].

Earlier studies showed that HF management in primary care is far from optimal, with underprescription of angiotensin-converting enzyme inhibitors (ACEIs) (or in the case of intolerance, angiotensin receptor blockers (ARBs)) and beta-blockers [7–10]. These studies did not, however, report data separately for patients with HFrEF and HFpEF. Moreover, most of these studies performed in primary care included patients with a GP’s diagnosis of HF, and thus a substantial number without a confirmatory echocardiogram, making it more likely that many false-positive HF cases were included [8–10].

Group education of GPs could possibly help improve the prescription of evidence-based drugs in, especially, HFrEF patients. Such education has effectively improved treatment in other primary care domains, e.g. proper antibiotic use and hypertension treatment [11, 12].

The primary aim of our study was to investigate whether a half-day training session for GPs on drug treatment of HF according to current guidelines combined with an easy-to-use uptitration leaflet to be used in clinical practice (see Electronic Supplementary Material) would improve drug therapy in HF patients, notably those with HFrEF.

## Methods

### Study population

Thirty primary care practices (PCPs), including urban, suburban and rural practices, located in the vicinity of Utrecht in the central Netherlands, participated in this study. The study was executed between November 2010 and March 2013. Approximately 70,500 patients were registered in these practices, with an average of 2350 per practice. Of note is the fact that every individual in the Netherlands, except for patients in nursing homes and hospices, is registered with a single GP, independent of specialist care, and GPs routinely register all patient contacts in an individual electronic medical record (EMR) and keep record of all specialist letters, including hospital discharge letters.

Eligibility criteria included men and women of 18 years or older and at least two documentations of HF in the patient’s EMR (International Classification of Primary Care (ICPC) code K77). Two documented codes were required, because we wanted to exclude accidental miscoding. In total, 683 patients were eligible. For this trial we included only the 398 (58.3%) patients in whom HF was confirmed by an expert panel [two cardiologists (AM and MAL) and a GP experienced in HF (FHR)] based on available data from cardiology hospital admissions, or outpatient visits and echocardiography.

The study was conducted according to the principles of the current version of the Declaration of Helsinki and in accordance with the Dutch Medical Research Involving Human Subjects Act (WMO). The study was approved by the Regional Medical Ethics Committee (MEC-U) of the Meander Medical Centre, Amersfoort, The Netherlands. All participating GPs gave written informed consent. All patients who filled out health status questionnaires gave written informed consent.

### Definition of HF by the expert panel

All relevant medical information on the eligible 683 participants was extracted from the EMRs and evaluated by an expert panel to determine the presence or absence of HF (the reference standard). Patients were considered to have definite HF when they met the criteria of the European Society of Cardiology (ESC), i.e. symptoms or signs suggestive of HF and objective echocardiographic evidence of a structural or functional abnormality of the heart at rest. The panel subdivided those with HF into HFrEF, HFpEF, or isolated right-sided HF; HFrEF if a patient’s left ventricular ejection fraction (LVEF) was ≤45% and HFpEF if a patient had a LVEF >45%, in the presence of at least two structural or functional abnormalities on echocardiography at rest (diastolic dysfunction). We considered as abnormal (1) a left atrial volume index (LAVi) >34 ml/m^2^, (2) E/e’ >15, (3) E/A <0.75 and (4) left ventricular wall thickness >11 mm. In patients with atrial fibrillation, a LAVi >34 ml/m^2^ was considered sufficient for the diagnosis of diastolic dysfunction.

Isolated right-sided HF was considered present if patients with suggestive HF symptoms had a calculated peak pulmonary artery pressure >40 mm Hg, without clear left ventricular dysfunction (arbitrary in our study, a LVEF >45%). Disagreement between panellists was resolved by majority of votes after discussion.

### Intervention

The 30 PCPs were allocated randomly to the intervention group or the care-as-usual (control) group. The GPs from the 15 PCPs allocated to the intervention group underwent a half-day group-training session on the diagnosis and drug treatment of HF based on recommendations of the most recent ESC HF guidelines [13]. Special attention was paid to differences in evidence-based drug treatment of patients with HFrEF and HFpEF. For patients with HFpEF, GPs were instructed to manage fluid retention with diuretics, control blood pressure and lower heart rate in the case of tachycardia (usually atrial fibrillation). GPs were instructed to treat patients with HFrEF with diuretics in the case of fluid retention and to uptitrate patients to maximally tolerated doses of an ACEI (or ARB if the ACEI was not tolerated) and a beta-blocker. In those patients with persistent symptoms (New York Heart Association (NYHA) class II or higher), GPs were instructed to additionally prescribe a mineralocorticoid receptor antagonist (MRA) [3]. GPs received an uptitration leaflet to assist them with careful uptitration of ACEIs and beta-blockers in daily practice. The GPs allocated to the control group did not receive the training or the uptitration leaflet. The study protocol has been published in detail elsewhere [14].

### Measurements

Baseline characteristics of participants were gathered from the EMRs of the 30 PCPs and included gender, age, comorbidities, date of HF diagnosis, drug prescriptions and results from echocardiography and natriuretic peptide measurements. Also noted was if participants received cooperative care from a cardiologist, defined as contact with the cardiology outpatient clinic or hospitalisation with admission to a cardiology ward in the previous 1.5 years. HF medication regimens were extracted from the EMRs 6 months after the training session (T1). Patients filled out two questionnaires on health status (the 36-Item Short Form Health Survey (SF-36) and the European Quality of Life Five Dimensions questionnaire (EQ-5D)) 12 months after the training session (T2). The SF-36 measures the health status of individuals with different health conditions in the following eight domains: physical functioning, bodily pain, general health perceptions, vitality, social role functioning, emotional role functioning, physical role functioning and mental health. Scores range from 0 to 100. The EQ-5D is a generic questionnaire that uses a visual analogue scale and provides a single index value for health status. It comprises five entities: mobility, self-care, usual activities, pain or discomfort, and anxiety or depression. Scores range from 1 to 3. Data on hospitalisations and mortality were obtained 28 months after the training session (T3).

### Outcomes

The primary outcome was the use of guideline-recommended HF medication in patients with HF at 6 months (T1). Secondary outcomes were health status at 12 months (T2) and mortality and hospitalisations (number of hospitalisations and number of hospitalisation days) at 28 months (T3).

### Statistical aspects

The sample size calculation was based on the changes in prescription rates in HFrEF patients. For the assumption of 30% beta-blocker use at baseline in patients with HFrEF, we used the results of a pilot study executed in our study region (Amersfoort, The Netherlands) among six GP practices covering 15,000 patients; in patients labelled with HF by the GP, the prescription rate of beta-blockers was 30%. Two studies executed in primary care found 36.6% and 38% beta-blocker use among patients with HF: the first study among ‘all-type HF’ and the other among HFrEF patients [15, 16].

We assumed the beta-blocker uptake would increase to 60% in 6 months in HFrEF patients from baseline, and that the level would remain at 30% in the control group. Based on these assumptions, 45 HF patients were required in each group to detect a 30% difference in prescription rates of beta-blockers, with an alpha of 0.05 and a power of 0.80, and 47 HFrEF patients in each group if we applied an intra-cluster correlation coefficient of 0.05 and a cluster size of 5. Considering a dropout rate of 10%, 52 HFrEF patients in each group were required. We calculated that approximately 30 PCPs would be needed to ensure that in total 104 patients with HFrEF were recruited [14].

Logistic regression analysis was used to estimate the training effect (the intervention) by calculation of the differences in HF drug use at 6 months (T1) between the intervention and control group corrected for use at baseline. Initially, we incorporated a random intercept in the logistic regression analysis to correct for clustering within PCPs. This clustering adjustment, however, showed no or very limited impact of clustering (σ^2^ ~ 0). We therefore applied ‘standard’ logistic regression without correction for clustering. Quality of life measured with the EQ-5D was analysed with a Mann-Whitney test.

Linear mixed-regression analyses, adjusted for baseline SF-36 scores and corrected for potential clustering in PCPs, were used to compare SF-36 scores of the control and intervention groups. Patients who died or were lost to follow-up in the period before the actual start of the study, i.e. between data extraction (January 2010) and the training session (October 2010), were excluded from the analysis. The mean number of hospitalisations and days of hospitalisation before 28 months (T3) were compared between the two groups using either Student’s *t*-test or Mann-Whitney U test. Kaplan-Meier survival curves were created to compare survival of HFrEF and HFpEF patients between the two groups over the 28-month period.

It was decided to include the five patients with isolated right-sided HF (four in the intervention group, one in the control group) in the HFpEF group before any statistical analysis was performed.

Statistical analyses were performed using SPSS version 20.0.

## Results

**Fig. 1:**
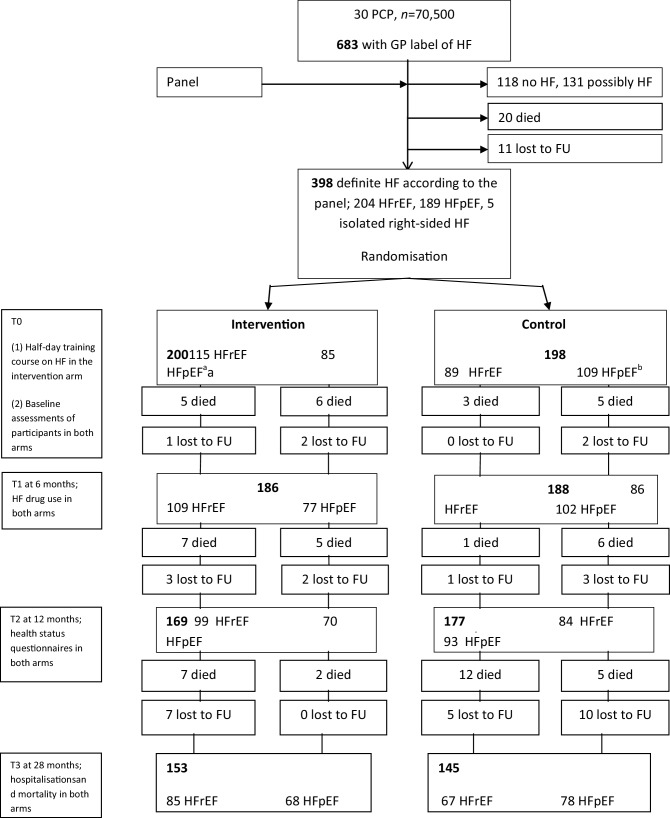
Flow chart. *PCP* primary care practice, *GP* general practitioner, *HF* heart failure, *HFrEF* heart failure with reduced ejection fraction, *HFpEF* heart failure with preserved ejection fraction, *FU* follow-up, *T0* time point of the training session of the intervention group and baseline assessments; *T1* at 6 months, assessment of the use of HF drugs; *T2* at 12 months, health status questionnaires; *T3* at 28 months, assessment of hospitalisations and mortality. ^a^ This group also included four patients with isolated right-sided HF. ^b^ This group also included one patient with right-sided HF

In total, 398 patients fulfilled the criteria of definite HF: 204 (51.3%) with HFrEF, 189 (47.5%) with HFpEF and 5 (1.3%) with isolated right-sided HF (Fig. 1). Mean age of the participants was 76.9 (SD 10.8) years, and 47.5% were male (Tab. 1). Prescription of evidence-based HF drugs in patients with HFrEF did not change significantly between baseline and T1 when comparing the intervention and control group (Tab. 2). At baseline, the use of beta-blockers was 59.1% in the intervention group and 60.7% in the control group. This increased by 5.2% [95% confidence interval (CI) 2.0–10.0] in the intervention group compared with 1.1% (95% CI 0.2–6.3) in the control group (baseline-corrected odds ratio (OR) 0.82 (95% CI 0.42–1.61)). At baseline, ACEI/ARB use was 68.7% in the intervention and 71.9% in the control group. It decreased by 5.2% (95% CI 2.0–10.0) in the intervention group compared with 5.6% (95% CI 2.8–13.4) in the control group [baseline-corrected OR 1.07 (95% CI 0.55–2.08)]. In HFpEF patients, there were also no clear differences in prescription rates of HF drugs between the two groups (Tab. 3).

**Table 1:** Baseline characteristics of 398 patients with established heart failure (*HF*) categorised per intervention and control groups, and per heart failure with reduced ejection fraction (*HFrEF*) and heart failure with preserved ejection fraction (*HFpEF*)

	Intervention group (*n* = 200)		Control group (*n* = 198)	
	HFrEF (*n* = 115)	HFpEF (*n* = 85)	HFrEF (*n* = 89)	HFpEF (*n* = 109)
Mean age, years (SD)	75.9 (11.1)	79.6 (7.9)	72.5 (12.8)	79.3 (9.2)
Male sex	59.1	40.0	55.1	34.9
Known to have HF, years (SD)	3.5 (3.0)	2.5 (2.4)	3.5 (3.7)	2.4 (2.6)
Cooperative care from cardiologist	73.9	57.6	73.0	55.9
Prior myocardial infarction	45.2	25.9	49.4	11.0
Angina pectoris	14.8	28.2	13.5	19.3
Atrial fibrillation	34.8	64.7	37.1	58.7
Stroke	8.7	18.8	12.4	15.6
Hypertension	40.9	61.2	53.9	68.8
Diabetes mellitus	35.7	28.2	29.2	36.7
COPD	25.2	22.4	15.7	17.4
eGFR <60 ml/min per 1.73 m^2^	40.9	43.5	38.2	41.3
Natriuretic peptides measured^a^	41.7	58.8	50.6	45.9

^a^Natriuretic peptides measured 10 months before T0

Numbers are percentages unless mentioned otherwise. Natriuretic peptide measurements were assessed in the 10 months before baseline. For baseline HF drug use see Tab. 2 and 3. Five patients (4 in the intervention group; 1 in the control group) with right-sided HF were counted as HFpEF. *COPD* chronic obstructive pulmonary disease, *eGFR* estimated glomerular filtration rate

**Table 2:** Proportion of prescribed heart failure (HF)-related drugs at baseline (*T0*) and after 6 months (*T1*) for the 204 patients with HF with reduced ejection fraction, divided into the intervention and control groups with baseline-corrected odds ratios (*bcORs*)

	Intervention arm (*n* = 115)					Control arm (*n* = 89)					
	T0	T1				T0	T1				bcOR (95% CI)
			Start	Continued use	Stop			Start	Continued use	Stop	
Diuretic	80.9	80.9	9.6	71.3	9.6	73.0	71.9	11.2	60.6	12.4	0.68 (0.33–1.39)
ACEI/ARB	68.7	63.5	10.4	53.0	15.7	71.9	66.3	6.7	59.6	12.4	1.07 (0.55–2.08)
BBeta-blocker	59.1	64.3	15.7	48.7	10.4	60.7	61.8	10.1	51.7	8.9	0.82 (0.42–1.61)
MRA	28.7	31.0	10.4	22.6	6.1	32.6	33.7	6.7	27.0	5.6	0.85 (0.39–1.88)

Numbers are percentages unless mentioned otherwise

*ACEI* angiotensin-converting enzyme inhibitor, *ARB* angiotensin receptor blocker, *CI* confidence interval, *MRA* mineralocorticosteroid-receptor antagonist, *T0* at baseline, *T1* after 6 months

**Table 3:** Proportion of prescribed heart failure (HF)-related drugs at baseline (*T0*) and after 6 months (*T1*) for the 194 patients with HF with preserved ejection fraction, divided into the intervention and control groups with baseline- corrected odds ratios (*bcORs*)

	Intervention arm (*n* = 85)					Control arm (*n* = 109)					
	T0	T1				T0	T1				bcOR (95% CI)
			Start	Continued use	Stop			Start	Continued use	Stop	
Diuretic	70.6	70.6	14.1	56.5	14.1	74.3	77.1	16.5	60.6	13.8	1.36 (0.70–2.65)
ACEI/ARB	52.9	57.6	20.0	37.6	15.3	55.0	55.0	14.7	40.4	14.7	0.86 (0.46–1.58)
Beta-blocker	62.4	56.5	10.6	45.9	16.5	49.5	52.3	14.7	37.6	11.9	1.09 (0.57–2.09)
MRA	24.7	17.6	5.9	11.8	12.9	25.7	28.4	11.0	17.4	8.3	2.18 (0.97–4.90)

Numbers are percentages unless mentioned otherwise

*ACEI* angiotensin-converting enzyme inhibitor, *ARB* angiotensin receptor blocker, *CI* confidence interval, *MRA* mineralocorticosteroid-receptor antagonist, *T0* baseline, *T1* after 6 months

**Fig. 2:**
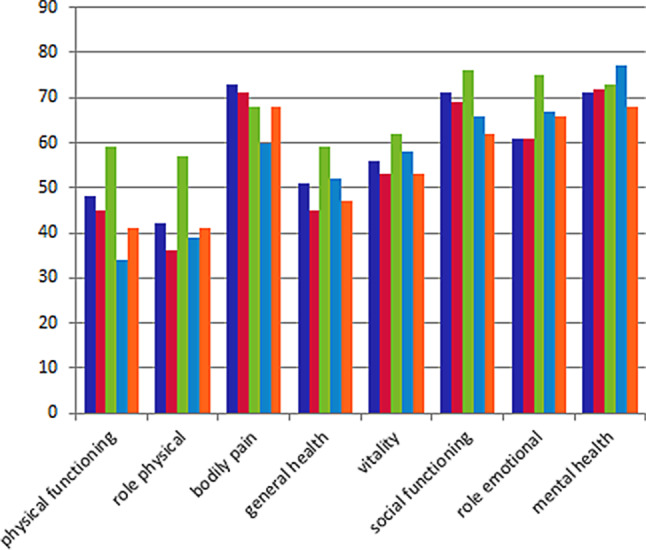
Health status assessed at 12 months (*T2*) with the 36-Item Short Form Health Survey and based on 96 patients with heart failure with reduced ejection fraction (HFrEF) and 68 patients with heart failure with preserved ejection fraction (HFpEF) in the intervention and control groups compared with data from the general population ≥70 years of age. *Blue* intervention group HFrEF, *red* control group HFrEF, *green* general population 70 years or over, *light blue* intervention group HFpEF, *orange* control group HFpEF

After 12 months, 38 patients had died (23 in the intervention and 15 in the control arm) and 14 were lost to follow-up (8 in the intervention group, 6 in the control group). Of the remaining 346 participants, 166 (48.0%) filled out the health status questionnaires. There were no statistically significant or clinically important differences [17] in the eight domains of the SF-36 scale between the intervention and control groups for patients with either HFrEF or HFpEF (Fig. 2). The EQ-5D did not show a significant difference in the five dimensions between the intervention and control groups, nor was a clinically important difference observed (data not shown). Tab. 4 shows the mean number of hospitalisations per year and the mean number of hospitalisation days per year for the two groups after a follow-up period of 28 months. At that time point, a total of 32 patients had died in the intervention arm and 32 patients in the control arm, and the numbers lost to follow-up were 15 and 21 in the intervention and the control arm, respectively. The mean number of hospitalisation days per year for patients with HFrEF in the intervention and control groups was 2.7 days/year and 2.1 days/year (*p* = 0.58), respectively. Cardiology hospitalisations were 1.0 day/year and 1.1 days/year (*p* = 0.22), respectively. Survival during 28 months did not significantly differ between the intervention and control groups for either HFrEF or HFpEF. Nineteen patients with HFrEF in the intervention group died compared with 16 in the control group (*p* = 0.72), while 13 patients with HFpEF in the intervention group died compared with 16 in the control group (*p* = 0.88).

**Table 4:** Mean number of hospitalisations per year and hospitalisation days per year during 28 months follow-up of the 398 patients divided into the intervention and control groups and per heart failure with reduced ejection fraction (*HFrEF*) and heart failure with preserved ejection fraction (*HFpEF*) patients, measured for all types of hospitalisation and cardiology hospitalisations separately

	Intervention group	Control group	*p*-value
*Mean number of hospitalisations/year*			
– All hospitalisations			
For HFrEF patients per year	0.4	0.3	0.31
For HFpEF patients per year	0.3	0.3	0.99
– Cardiology hospitalisations			
For HFrEF patients per year	0.2	0.2	0.20
For HFpEF patients per year	0.1	0.1	0.79
*Mean number of all-type hospitalisation days/year*			
For HFrEF patients	2.7	2.1	0.58
For HFpEF patients	2.2	2.1	0.84
– Cardiology hospitalisations			
For HFrEF patients per year	1.0	1.1	0.22
For HFpEF patients per year	0.3	0.7	0.90

Four patients with isolated right-sided HF were included in the intervention group. One patient with isolated right-sided HF was included in the control group

## Discussion

In this cluster randomised controlled trial among 398 patients with established HF, a half-day GP training session on diagnosis and drug treatment of HF did not improve drug treatment or clinical outcomes of patients with either HFrEF or HFpEF.

Among the 204 patients with HFrEF, the use of ACEIs/ARBs decreased by 5.2% (95% CI 2.4–10.9) in the intervention group and by 5.6% (95% CI 2.4–12.5) in the control group [baseline-corrected OR 1.07 (95% CI 0.55–2.08)], while beta-blocker use increased by 5.2% (95% CI 2.0–10.0) in the intervention group and 1.1% (95% CI 0.2–6.3) in the control group [baseline-corrected OR 0.82, (95% CI 0.42–1.61)].

These neutral results may be explained by several mechanisms. A half-day training session and an uptitration leaflet were insufficient to affect GPs’ prescription behaviour—more so in patients with established HF and known to have had this disease for 3.0 (SD 3.0) years on average, and with collaborative care from the cardiologist in 65% of the cases. Moreover, selecting patients with established HF among those labelled with HF in primary care has played a role in the higher baseline treatment uptake of ACEIs/ARBs and beta-blockers than we assumed for our power calculation, which was based on a pilot study in the area and two previous studies performed among patients with a GP’s diagnosis of HF [15, 16]. In all three of these studies beta-blocker uptake was between 30% and 40%.

A recent Dutch study executed in 34 Dutch HF outpatient clinics (10,910 patients with HF; 5701 (52.3%) with a LVEF <40%) showed that in these routine care patients with HFrEF (with LVEF <40%) 81% were on loop diuretics, 84% on renin-angiotensin-system inhibitors, 86% on beta-blockers and 56% on MRAs [18]. Thus, there seems to be ample room for improvement in the routine care of patients with HFrEF, in whom the HF care is more or less orchestrated by GPs.

We kept the training course simple and pragmatic with a focus on implementation of evidence-based effective treatment in those with HFrEF. A more intensive training course or, probably even better, combined training with cardiologists and HF nurses may have achieved better results. The complexity of HF management demands a multidisciplinary team approach, not only while the patient is hospitalised and initially uptitrated, but also during the more stable chronic phase of the disease, because the disease trajectory of HF is also characterised by the development of new (non-cardiac) comorbidities, which may affect the tolerance to HF drugs.

Importantly, two previous studies showed that patients with HFrEF could, after initially having been optimally uptitrated with HF drugs at the outpatient cardiology clinic, be monitored equally effectively and safely in primary care with regard to guideline adherence and patient adherence [19, 20]. HF drug therapy changes, e.g. starting and stopping HF medication, were equal in HF clinic and GP care in the study of Schou et al. [20]. In the study of Luttik et al. no differences were observed in drug adherence between patients allocated to continuation of HF care at the cardiology outpatient clinic or to monitoring in primary care [19]. The patients in this Dutch study were known to have had HF for 3 years on average (i.e. comparable with the patients in our study).

Gupta and colleagues suggested in 2004 that it should be possible to adequately uptitrate beta-blockers in up to 70% of patients with HFrEF, taking into account (relative) contraindications, old age and other drugs [21]. However, the current results of the CHECK-HF study suggest that even 86% seems possible in routine care [18].

In 2008, a German study showed that GPs were not able to further uptitrate HFrEF patients who were already on a high beta-blocker prescription rate (79%), even after the GPs had received a very intensive training programme (in total 16 h) [22]. In 2009, Calvert and co-workers reported that 36.6% of patients known to have ‘all-type’ HF in primary care received beta-blockers, and 29.3% ACEIs/ARBs; however, they did not provide the findings for HFrEF and HFpEF separately [15]. In a Spanish paper published in 2010, it was shown in a randomised study that GPs who attended a simple single interactive training session managed to prescribe beta-blockers to a higher proportion of patients with HFrEF than GPs who did not receive such training (49% optimal tolerated dose within 3 months vs 38% in the usual care group) [16].

At the time of our study, angiotensin receptor-neprilysin inhibitors were not yet available in the Netherlands for routine care, so these could not be studied.

Since there is no available pharmacological treatment that clearly reduces morbidity and mortality in patients with HFpEF, the lack of an effect on HF drug prescription rates in HFpEF was not surprising, albeit we could have expected some effect on MRA prescription, because a post hoc subgroup analysis of the TOPCAT study recently suggested that spironolactone may have a beneficial prognostic effect in HFpEF patients with a LVEF >45% [23, 24]. In contrast, however, the prescription of MRAs in HFpEF patients in the intervention group of our study was reduced (Tab. 3).

The SF-36 scores 12 months after the training session in our study for patients with HF (HFrEF and HFpEF) were comparable with the results on the health domains at 12 months as reported by Holzapfel et al. [25], Juenger et al. [26] and Scherer et al. [27] Those three studies compared the 1‑year SF-36 scores with baseline health status scores, and showed that there were only small differences that were considered clinically unimportant. In our study population, all indices of health status were lower than those in ≥70-year-old community-dwelling men and women without HF studied by Aaronson et al. [28], with the most pronounced differences being the domains of physical functioning, role physical and role emotional. Similar differences between patients with HF and the population at large were found in earlier studies [27, 29, 30]. One study performed in Russia reported on an intensive nurse-led care programme in primary care, focusing on lifestyle changes and modification of cardiovascular risk factors, exercise training and intensive proactive nursing care in 85 patients with HFpEF. After 6 months of follow-up health improved in the intervention arm compared to usual primary care. However, the quality of usual primary care of HF patients in Russia is very likely lower than in the Netherlands, thus leaving more room for improvement following an intervention in the primary care setting in Russia [31]. Importantly, cardiovascular mortality and readmissions rate were not reduced in the Russian study [31].

Of the 683 patients with a GP label of HF, a panel could establish HF in only 398 (58.3%) patients, mainly because of insufficient diagnostic work-up with the lack of data from electrocardiography, N‑terminal pro-brain natriuretic peptide (NT-proBNP) or echocardiography. Thus, GPs did not adequately follow the recommendations of the 2010 Dutch GP guidelines recommending the measurement of NT-proBNP and recording of an electrocardiogram (ECG) in patients suspected of HF based on signs and symptoms [32]. The main strength of our study lies in the study population, which was a representative sample of the general population of patients with established HF in the Netherlands. We had access to all data on medication, cardiologist letters, hospitalisations and death. Moreover, an expert panel confirmed the presence or absence of HF in potential participants.

### Limitations

Our power calculation was based on the assumption that 30% of patients with HFrEF would be prescribed beta-blockers at baseline. However, this assumption was too low (eventually 60% were on beta-blockers at baseline), and based on a pilot and data from the literature mainly considering ‘all-type’ HF patients, thus including patients with HFpEF [15, 16].

Because we included patients with established HFrEF with an average duration of HF of more than 3 years, and with 70% in cooperative cardiology care, it was over-optimistic to speculate on improved HF drug uptake with just a half-day training session for GPs.

Only a limited number (48.0%) of health status questionnaires were filled out completely. Moreover, SF-36 scores were analysed after adjustment with imputed baseline SF-36 scores, but without correction for clustering. However, as there was no intervention effect, not correcting for clustering is very unlikely to have influenced the results.

A more detailed assessment of changes in drug prescription, including changes in dosages and registration of the defined daily dose (DDD) would be more informative, but would also result in much higher costs in performing the study. The lack of DDD also prevented us from calculating the number of patients with HFrEF on the recommended HF dosage.

Finally, the follow-up period was relatively short for detecting any change in medication.

Systematic reviews of the literature focused on implementation strategies identified four successful strategies for getting research into practice: computerised decision support, opinion leaders, financial incentives and audit-and-feedback [33]. Combinations of these were more effective than a single approach. Such strategies, but also a multifaceted approach in which GPs are trained together with cardiologists and HF nurses, are possibly the best option to optimise HF management. The Dutch CONNECT-HF trial, in which HF care is organised regionally, is a very helpful initiative to improve patient-centred HF management.

## Conclusions

A half-day training programme for GPs does not improve HF drug prescriptions in patients with established HF. Other interventions, such as a multidisciplinary approach, should be considered for optimising HF drug treatment in stable HF patients primarily managed in primary care.

## Caption Electronic Supplementary Material

Leaflet uptitration HF medication. HFrEF, and HFpEF
