# Supplementary figures and images for: Training general practitioners to improve evidence-based drug treatment of patients with heart failure: a cluster randomised controlled trial
Source: Neth Heart J. 2020 Sep 30;28(11):604–12. doi: 10.1007/s12471-020-01487-x (PMC7596131; doi:10.1007/s12471-020-01487-x)

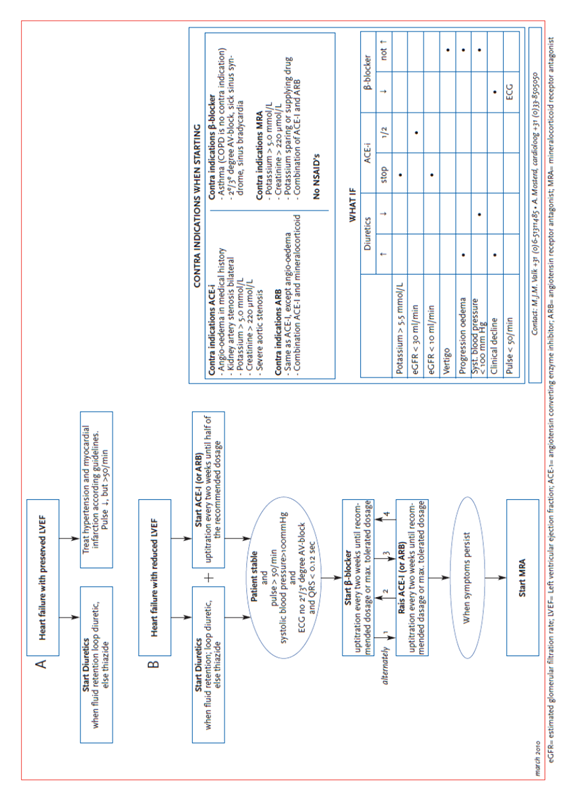


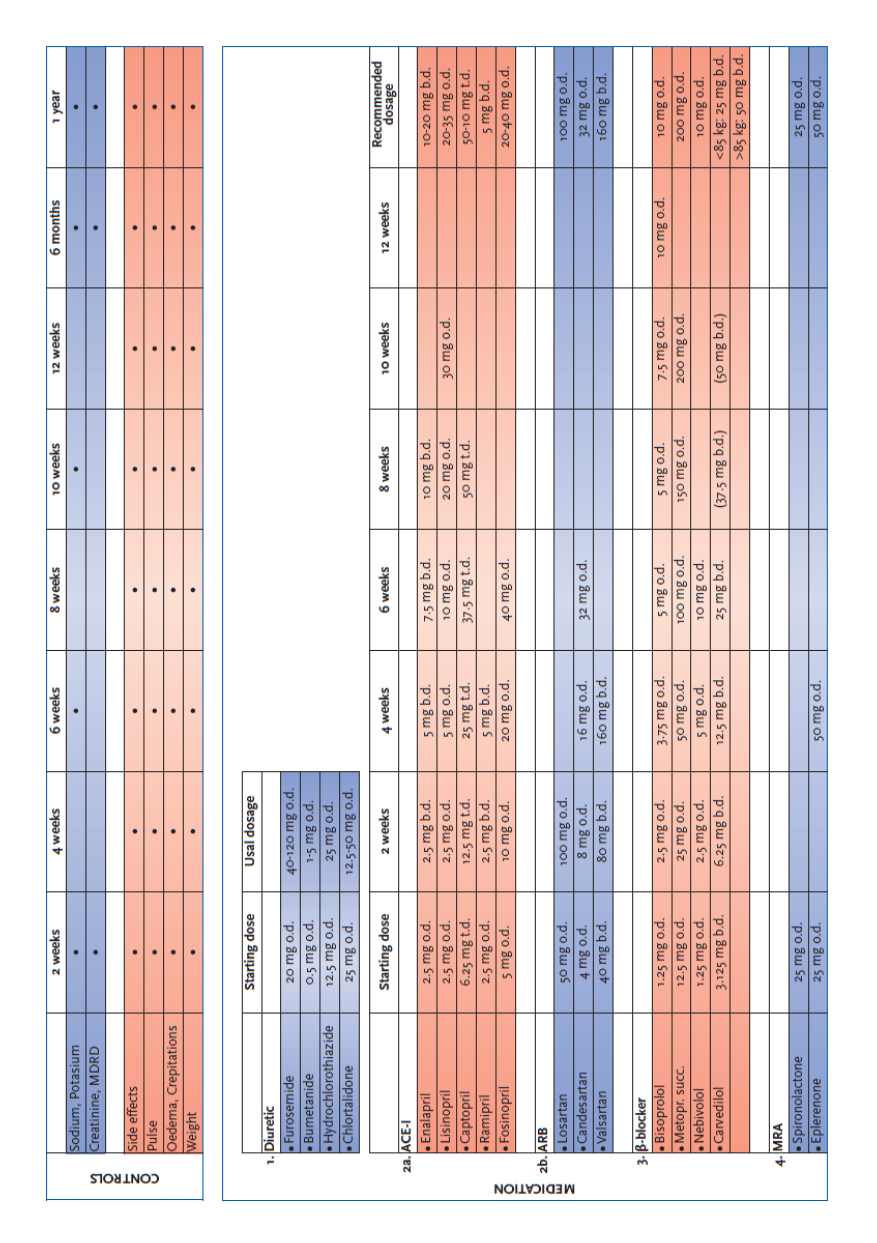

Supplement: Supplementary file 1 — Leaflet uptitration HF medication. HFrEF, and HFpEF [file 12471_2020_1487_MOESM1_ESM.docx]
